# Naturalistic generative narratives reveal effects of social characteristics on decision-making

**DOI:** 10.3389/fpsyg.2024.1412131

**Published:** 2024-11-18

**Authors:** Ethan Wong, Ofir Williams, Ziv M. Williams, Raymundo Báez-Mendoza

**Affiliations:** ^1^Department of Neurosurgery, Massachusetts General Hospital and Harvard Medical School, Boston, MA, United States; ^2^Northeastern University, Boston, MA, United States; ^3^Health Sciences and Technology, Harvard-MIT, Boston, MA, United States; ^4^Program in Neuroscience, Harvard Medical School, Boston, MA, United States; ^5^Department of Neurobiology, German Primate Center, Göttingen, Germany

**Keywords:** explicit attitude, decision making, contrast effect, social context, sociodemographic, emotional state, socioeconomic status, generative narrative survey

## Abstract

**Introduction:**

The social characteristics of others can powerfully influence our decisions. They can also be broadly impacted by the social context in which these choices are made, making the effects of these characteristics on decision-making especially challenging to understand.

**Methods:**

Here, we developed a Generative Narrative Survey that provided participants with naturalistic scenarios that richly varied in social context and theme but that also systematically varied the characteristics of the social agents involved, followed by a question. An example of this narrative is *“You’re a tourist, and you are trying to take a picture of yourself with your phone. A black male comes up to you and offers to take the photo for you. Do you hand them your phone?”*

**Results:**

After validating this approach using feeling thermometer measures, we found that the emotional states of others had the strongest and most consistent effect on the participants’ choices. More notably, whereas most characteristics had independent effects on decision-making, social features such as the inferred socioeconomic status of others significantly influenced the effect that race had on the participant’s judgments. Moreover, the social context of the agent’s interactions with other agents had a significant additive effect, especially when the emotional states of the agents in the scenarios contrasted. The influence of these characteristics on the participants’ choices was also markedly affected by their demographics, especially when contrasting with that of the agents involved, and were often driven by the participants’ reported political views.

**Discussion:**

Together, these findings reveal how the mixture of social characteristics, context, and personal views influence decision-making and highlight the use of naturalistic generative narrative surveys in studying human behavior.

## 1 Introduction

The social characteristics of others can powerfully influence our decisions (Cunningham and Zelazo, 2007; Fiske and Taylor, 2013). Negative attitudes, for example, are often associated with avoidance, ignoring others, and selective re-interpretation, while positive attitudes are often associated with approaching, selective attention, and preferential information recall. Thus, the evaluation of different characteristics and the context in which the evaluation takes place can profoundly affect how we interpret different situations we face in real life.

Explicit attitudes are pervasive and can be based on another’s race, gender, socioeconomic status, and perceived emotions (Axt, 2018; Charlesworth and Banaji, 2019; Fitzgerald and Hurst, 2017; Kurdi et al., 2019a; Payne et al., 2008). Measures of explicit attitudes often ask participants to express their evaluations deliberately in the form of a survey (Payne et al., 2008), these surveys typically focus on single characteristics such as age or race, but not on multiple characteristics. People are not always aware of their attitudes or might have implicit biases in their evaluation of distinct characteristics (Greenwald and Banaji, 1995). Thus, implicit attitude tests have also been used to reveal people’s implicit attitudes, yet, these tests are rarely presented within the context of real-world scenarios or social contexts and typically focus on contrasting two characteristics at the time.

Although there has been significant progress in our understanding of how explicit and implicit attitudes affect our perception of others (Daumeyer et al., 2019), more needs to be done to understand how these biases may explicitly influence our decision-making. More importantly, more needs to be understood about how our decisions are affected by the complex interaction between traits such as race, gender, and personality. For instance, are certain decisions more likely to be negatively affected if one agent is black and friendly versus black and angry or a well-dressed female versus a poorly-dressed male? We also need to know more about how one’s demographics influence these decisions.

Finally, while an individual’s characteristics may influence our decisions, they can also be strongly impacted by the social context in which they are made (Allen et al., 2010). For example, people compare their attitudes to those of others and might even adjust them based on their perceived similarities (Adolphs, 2003; Heider, 1958; Hovland et al., 1957) or the number of agents that are involved (Bower, 1961). Further, the setting in which interactions between two agents occur and how they manifest can affect our judgment of those individuals (Wang et al., 2023; Yang et al., 2023). While attitudes are often prone to contrast effects (Hovland et al., 1957) and ingroup beliefs (Efferson et al., 2008), these ingroup preferences and outgroup dislikes can also be reflected in our demographics, including our political leaning (Leshin et al., 2022). Thus, contrast effects, ingroup favoritism, and political preferences provide additional rich contexts that can influence attitudes.

Here, we aimed to study how the mixture of social characteristics, contexts, and personal beliefs influence our decisions by developing a Generative Narrative Survey design. We refer to our approach Generative Narrative, as it generates distinct narrative items on every survey based on a set of rules. Specifically, we aimed to determine how different permutations of social characteristics of others (e.g., emotional state, perceived socioeconomic status, race, and gender), their context (e.g., the interaction between social agents), and participant’s demographics may affect participant’s decisions, and how combinations of these factors interact to produce choices across scenarios that are generalizable and robust.

## 2 Methods

### 2.1 General design

The experiments were approved by the Ethics Committee of the Georg-Elias-Müller Institute of Psychology of the University of Göttingen. For the study, we developed a Generative Narrative Survey in which a series of brief written scenarios were provided to participants. The participants were instructed to answer a series of questions. Specifically, *“For each question, we ask you to imagine yourself in a real-life scenario. In every scenario, you will observe or interact with others. Please answer questions as true to yourself as possible.”* The scenarios present situations in which one or two agents take an action. The agents in the scenarios possess two to three different characteristics describing their emotional state, socioeconomic status, race, and gender. To allow for tractable analysis across different combinations of characteristics, each category of characteristics could take two values. For emotion, it was ‘happy’ or ‘angry’; for socioeconomic status, it was ‘poorly-dressed’ or ‘well-dressed’, for race it was ‘white’ or ‘black’; for gender, it was ‘female’ or ‘male.’ After each scenario, the participants were prompted to answer a question deciding between two, three, or four alternatives.

### 2.2 Generative narrative survey generation

We generated a unique survey for each participant. It had eight demographic questions, fifty generated narratives, five comprehension questions, and eight feeling thermometer questions. The main test consisted of answering 50 items containing a ‘stem narrative’ in which one or two agents participated. A base set of 46 “template” narratives (Table 1) and 39 comprehension-specific “template” narratives (Table 2) were used in survey generation (7 “template” narratives did not have an obvious comprehension-specific equivalent, see Table 2). To allow for generalizability, we generated unique surveys for each participant in which they answered the same 50 narratives, but each narrative had agents with different characteristics. For example, they may be presented with the narrative *“You’re a tourist, and you are trying to take a picture of yourself with your phone. A black male comes up to you and offers to take the photo for you.”* We then asked the participants to indicate in a 2-, 3-, or 4-item response their judgment of each narrative. In this example, the question was “*Do you hand them your phone?*” Other scenarios, by comparison, may contain a narrative such as *“You are a juror. The defendant is a poorly-dressed female and, based on the facts, they likely stole millions of dollars.”* followed by the question *“How many years would you recommend in jail?”* Further, these scenarios would randomly vary such that sometimes there would be a white male in one narrative and a white female in another, or a happy male in one and an angry male in another.

**Table 1:** Template narratives, question, answers, and assigned scoring.

No	Narrative	Question	Answers	Scoring
1	You have just finished eating at a nice restaurant. Your waiter, who is a %s, was very slow in delivering your food.	How much would you tip them?	0, 10, 20%	0, 0.5, 1
2	You had a terrible cab ride home from the airport, driven by a %s.	How much would you tip them?	0, 10, 20%	0, 0.5, 1
3	Your boss at the office is interviewing two candidates for a job. The first one is a %s and the second one is a %s.	Who do you think will be hired?	First, Second	1, 0;0, 1
4	You’re a beggar on the streets of a major city. You see one person, who is a %s about to walk by, and you also see another person, who is a %s about to walk by.	Who do you ask for money?	First, Second	1, 0;0, 1
5	You are walking on the street and see a %s lying on the side of the street. They have a sign asking for money and seem homeless and hungry.	How much money would you give?	$0, $5, $20	0, 0.5, 1
6	You are walking on the street and a %s asks you for money. They seem homeless and hungry.	How much money would you give?	$0, $5, $20	0, 0.5, 1
7	As you are strolling in the street, you hear a %s yelling at a %s.	Do you intervene?	Yes, No	0, 1;1, 0
8	As you are walking in the mall, you hear a %s screaming at a %s.	Do you intervene?	Yes, No	0, 1;1, 0
9	You see a %s wearing very expensive shoes.	How do you think they got the shoes?	Stolen, Bought	0, 1
10	Two cars are speeding toward two persons a %s and a %s. You can only save one person.	Who do you save?	First, Second	1, 0;0, 1
11	You are at a supermarket about to leave. You hear a %s telling the cashier that they are 5 dollars short.	Do you offer them 5 dollars?	Yes, No	1, 0
12	You and your friends are playing basketball. A %s asks you to leave since they have been waiting for a while to play.	Do you leave?	Yes, No	1, 0
13	A %s approaches you saying that they lost their phone and need to make a phone call.	Do you let them borrow your phone?	Yes, No	1, 0
14	You are feeling hungry. You see two food trucks, the first with a %s and another with a %s handling and selling the food.	Which food truck will you eat at?	First, Second	1, 0;0, 1
15	You are thirsty. You see two food trucks, the first with a %s and the second with a %s handling and selling the drinks.	Which food truck will you buy a drink from?	First, Second	1, 0;0, 1
16	You are walking down the street and see two people wrestling. One is a %s and the other is a %s. They both look in pain.	Who do you think started the fight?	First, Second	0, 1;1, 0
17	You are strolling in the park and see two people wrestling. One is a %s and the other is a %s. They both look in pain.	Who do you think started the fight?	First, Second	0, 1;1, 0
18	You are a juror. The defendant is a %s and, based on the facts, they likely possessed a large amount of illegal drugs at their home.	How many years would you recommend in jail?	7 years, 15 years, 20 years	1, 0.5, 0
19	You are a juror. The defendant is a %s and, based on the facts, they likely stole millions of dollars.	How many years would you recommend in jail?	7 years, 15 years, 20 years	1, 0.5, 0
20	You are a bank officer. A %s approaches you for a $10,000 loan but they do not have good credit.	How much would you loan them?	$0, $5000, $10000	0, 0.5, 1
21	You inherited a large sum of money. A representative, a %s, from a local charity asks for a donation.	How much would you donate?	$0, $5000, $10000	0, 0.5, 1
22	You are a cab driver dropping off a %s. When you tell them that the fare is $20, they say that they forgot their wallet.	Do you believe them?	Yes, No	1, 0
23	You are picking up a %s passenger in your cab. They seem to be very dirty and smell of alcohol.	Do you let them into your cab?	Yes, No	1, 0
24	You are the manager of a grocery store and are looking to hire help. Two people apply for the job. The first is a %s and the other is a %s	Who would you hire?	First, Second	1, 0;0, 1
25	As the manager of a restaurant, you need to hire help. Two people apply for the job. The first is a %s and the other is a %s.	Who will you hire?	First, Second	1, 0;0, 1
26	You are a police officer who has just arrived at the scene of a robbery. Two people are walking out of the store. The first is a %s and the second is a %s.	Who would you question first?	First, Second	0, 1;1, 0
27	You are a police officer who has just arrived at the scene of a robbery. You see two people running. The first is a %s and the second is a %s.	Who would you question first?	First, Second	0, 1;1, 0
28	You are waiting in line to return empty plastic bottles to get $0.05 per bottle. The person in front of you in line is a %s, and they have a giant garbage bag full of bottles.	Where did they fill the bag?	Home, Dumpster	1, 0
29	You’re walking through a wealthy neighborhood and you see two individuals conversing on a large mansion’s terrace. The first is a %s and the second is a %s.	Which person do you think owns the house?	First, Second	1, 0;0, 1
30	You are a social worker waiting to meet a new client looking for help finding child support. You see two people in your waiting room: the one on your left is a %s and the one on your right is a %s.	Who do you think is your client?	Left, Right	0, 1;1, 0
31	You are browsing the internet for budgeting advice. Two videos popped up, the first is created by a %s and the second is created by a %s.	Which video do you watch for advice?	First, Second	1, 0;0, 1
32	You are a counselor at a school for all ages, and a teacher calls to tell you they send student to your office for cheating on a test. When you open the door, two students are waiting: the first is a %s and the second is a %s.	Who do you think was caught cheating?	First, Second	0, 1;1, 0
33	You’re at the train station and a %s approaches you asking for $5 to help them pay for their ticket home.	Do you give them money?	Yes, No	1, 0
34	You’re a barista at a coffee shop. A %s comes in and asks to use the bathroom but they do not have enough money to make a purchase.	Do you let them use the bathroom?	Yes, No	1, 0
35	You’re a tourist, and you are trying to take a picture of yourself with your phone. A %s comes up to you and offers to take the photo for you.	Do you hand them your phone?	Yes, No	1, 0
36	You’re a theater play director. You’re deciding between two candidates for the starring role. The first is a %s and the second is a %s.	Which person do you cast as the starring role?	First, Second	1, 0;0, 1
37	You walk into a classroom on the first day of class and there are two seats open. One seat is next to a %s and the other seat is next to a %s.	Which person do you sit next to?	First, Second	1, 0;0, 1
38	A local politician, who is a %s, is running for office.	Would you vote for this politician?	Yes, No	1, 0
39	A %s is eating lunch in the park.	Do you think they paid for the lunch?	Yes, No	1, 0
40	You are a famous sports personality. A %s asks for an autograph.	Do you give them an autograph?	Yes, No	1, 0
41	You are $10 short for your groceries. A %s offers you that amount.	Do you accept the money?	Yes, No	1, 0
42	You are driving home, and a %s asks for money in an intersection.	How much money do you give?	$0, $1, $5, $10	0, 0.3, 0.6, 1
43	You are a police officer investigating a crime in the neighborhood. You see a %s running on the street.	Do you suspect them of committing the crime?	Yes, No	0, 1
44	You are strolling on the boardwalk and hear and see a %s playing their guitar with their case open for money.	How much money do you place in their case?	$0, $5, $20	0, 0.5, 1
45	You’re at a soup kitchen and see a %s.	Do you think the individual is there to get food or to volunteer?	Get food, Volunteer	0, 1
46	There was a car accident involving two cars. Two drivers, a %s and a %s, are arguing.	Who do you think caused the accident?	First, Second	0, 1;1, 0

Narratives shown to the participants are constructed by assigning one or two adjectives and a noun to the placeholder “%s” in the narrative. If a narrative has two characters, at least one of the adjectives or one of the nouns was drawn from the same category. Scoring corresponds to the order of the possible answers. Values in scoring after the semi-colon correspond to the narrative scores for the second character in the narrative.

**Table 2:** Template comprehension narratives, questions, answers, and assigned scoring.

No	Narrative	Question	Answers	Scoring
1	A typical restaurant tip in the USA is 20%%. Your waiter, who is a %s, was very slow in delivering your food.	What is the typical restaurant tip?	0, 10, 20%	0, 0, 1
2	Cab rides in the USA are typically tipped at 10%%. You had a terrible cab ride home from the airport, driven by a %s.	What is the typical cab ride tip?	0, 10, 20%	0, 1, 0
3	You’re a security guard at a business office who is hiring new workers. You see the first candidate, a %s walking out of the office, and you see the second candidate, a %s walking out of the office.	What is your job?	Doorman, Security guard	0, 1
4	You’re a beggar on the streets of a major city. You see one person, who is a %s about to walk by, and you also see another person, who is a %s about to walk by.	Where are you?	Major city, Rural area	1, 0
5	You are walking on the street with a $20 bill in your pocket and see a %s lying on the side of the street. They seem homeless and hungry.	Do you have a $20 bill in your pocket?	Yes, No	1, 0
6	You are walking on the street and a %s asks you for $5. They seem homeless and hungry.	How much money did they ask for?	$0, $5, $20	0, 1, 0
7	As you are strolling in the park, you hear a %s receiving a racist remark from a %s.	Where were you walking?	Park, Beach	1, 0
8	As you are walking in the mall, you hear a %s receiving a racist remark from a %s.	Where were you walking?	Mall, Stadium	1, 0
9	While shopping at a store, you notice a %s taking an item from the shelf and hiding it in their jacket. They start heading toward the exit.	Did they hide the item in their jacket?	Yes, No	1, 0
10	While walking on the street, you notice a %s pickpocketing someone.	Are you driving your car?	Yes, No	0, 1
11	You see a %s crossing the street. A car is speeding toward them while running a red light.	Is the car running a red light?	Yes, No	1, 0
12	You are at a supermarket about to leave. You hear a %s telling the cashier that they are 5 dollars short.	Does the shopper need $15?	Yes, No	0, 1
13	You and your friends are playing basketball. A %s asks you to leave since they have been waiting for a while to play.	Are you playing volleyball?	Yes, No	0, 1
14	A %s approaches you saying that they lost their phone and need to make a phone call.	Do you let them borrow your computer?	Yes, No	0, 1
15	You are feeling hungry. You see two food trucks, the first with a %s and the second with a %s handling and selling the food.	How many food trucks were there?	One, Two	0, 1
16	You are thirsty. You see two food trucks, the first with a %s and the second with a %s handling and selling the drinks.	How many food trucks were there?	One, Two	0, 1
17	You are walking down the street and see two people fighting. The first is a %s and the other is a %s. The first looks in pain.	Where were you walking?	Park, Street	0, 1
18	You are strolling in the park and see two people fighting. The first is a %s and the other is a %s. The second looks in pain.	Where were you walking?	Park, Street	1, 0
19	You are a juror. The defendant is a %s and, based on the facts, they may be sentenced to 7 years in jail for their crime.	How many years will they be in jail?	7 years, 15 years, 20 years	1, 0, 0
20	You are a juror. The defendant is a %s and, based on the facts, they may be sentenced to 15 years in jail for stealing millions of dollars.	How many years will they be in jail?	7 years, 15 years, 20 years	0, 1, 0
21	You are a bank officer. A %s and %s couple approach you for a $10,000 loan but they do not have good credit.	For how much money did they ask in a loan?	$0, $5, 000, $10, 000	0, 0, 1
22	You want to donate $5,000. Two representatives, a %s and a %s, from the charity you care the most about approach you asking for a donation.	How many representatives were there?	One, Two	0, 1
23	You are a cab driver dropping off a %s. When you tell them that the fare is $40, they say that they forgot their wallet.	Are you driving a cab?	Yes, No	1, 0
24	You are picking up a %s passenger in your cab. They seem to be very dirty and smell of alcohol.	Are you driving a cab?	Yes, No	1, 0
25	You are the manager of a grocery store and are looking to hire help. Two people show up to interview, the first is a %s and the other is a %s.	How many people came to the interview?	One, Two	0, 1
26	As the manager of a restaurant, you need to hire help. Two people show up to interview, the first is a %s and the other is a %s.	How many people came to the interview?	One, Two	0, 1
27	You are a police officer who has just arrived at the scene of a robbery. You see a %s running and a %s walking. Both of them seem to have full pockets.	Who was running?	First, Second	1, 0
28	You are a police officer who has just arrived at the scene of a robbery. You see two people running. The first is a %s undercover police officer and the other is a %s.	Who do you chase?	First, Second	0, 1
29	You are waiting in line to return empty plastic bottles to get $0.05 per bottle. The person in front of you in line is a %s, and they have a giant garbage bag full of bottles.	How much is a bottle deposit?	0.25, 0.05	0, 1
30	You’re walking through a wealthy neighborhood and you see two individuals conversing on a large mansion’s terrace. The first is a %s and the second is a %s.	How many individuals were talking on the terrace?	Two, Three	1, 0
31	You are a social worker waiting to meet a new client looking for help finding child support. You have never met them, and see two people in your waiting room: the one on your left is a %s and the one on your right is a %s.	What does your client need help with?	Finding child support, Finding a job	1, 0
32	You are browsing the internet for budgeting advice. Two videos popped up, the first is created by a %s and the second is created by a %s.	What do you need help with?	Budgeting, Shopping	1, 0
33	You are a counselor at a school for all ages, and a teacher calls to tell you they send student to your office for cheating on a test. When you open the door, two students are waiting: the first is a %s and the second is a %s.	What do you do for work?	Schoolteacher, Counselor	0, 1
34	You are a teacher at a specialized school for people of all ages with behavioral difficulties. A student reports that “Alex” has been prank calling her, but you have two “Alexes” in your class: the first is a %s and the second is a %s.	What do you do for work?	Teacher, Physiotherapist	1, 0
35	You’re at the train station and a %s approaches you asking for $5 to help them pay for their ticket home.	Are you at the train station?	Yes, No	1, 0
36	You’re a barista at a coffee shop. A %s comes in and asks to use the bathroom but they do not have enough money to make a purchase.	Where do you work?	Coffee shop, Restaurant	1, 0
37	You’re a tourist, and you are trying to take a picture of yourself with your phone. A %s comes up to you and offers to take the photo for you.	What are you trying to do?	Take a photo, Get directions	1, 0
38	You’re the director of a theatrical performance at a local theater. You’re deciding between two candidates for the starring role. The first is a %s and the second is a %s.	How many candidates are you deciding between?	Two, Three	1, 0
39	You walk into a classroom on the first day of class and there are two seats open. One seat is next to a %s and the other seat is next to a %s.	Is it the first day of class?	Yes, No	1, 0

Narratives shown to the participants are constructed in the same way as for test narratives. Answers to the question are provided in the narrative and scored accordingly.

Each unique test was generated by pseudo-randomly assigning to each item two or three testable characteristics. If there were two agents in the narrative, then, at least one of their characteristics was complementary. No two narratives that were semantically similar or that included race as a characteristic were presented in succession. Comprehension questions were pseudo-randomly interspersed in the test and were not asked in succession.

Each generated survey included at least one instance of each of the 46 template narratives. To allow for generalizability and diversity of narratives, 20% of the narratives generated included two characteristics (e.g., white male) and 80% included three descriptors (e.g., poorly-dressed white male). If a narrative included two agents, the two agents always contained distinct descriptors from the same category. Within each survey, the narratives were generated so that each descriptor and combination of descriptors were featured in roughly equal numbers.

To confirm that the participants indeed attended to the questions and to test their comprehension, the survey included five narratives in which the question was directly related to the narrative. For example, “*You’re the director of a theatrical performance at a local theater. You’re deciding between two candidates for the starring role. The first is a white male and the second is a black male. How many candidates are you deciding between?*” Respondents with an accuracy below 60% were excluded from further analyses (*n =* 2 were excluded).

As an additional convergent validity measure, participants filled in a feeling thermometer after filling in the survey, in which participants rated their feelings toward each characteristic from “*very cold and unfavorable*” to “*very warm and favorable*.” Note that in both tests, the participants had to consider statements, evaluate their possible responses, and decide how to best express their responses (Payne et al., 2008).

Finally, the survey contained a series of demographic questions including year of birth, highest education achieved, self-identified race or races, self-identified gender, and household income based on the US census quartiles. Furthermore, based on the social capital hypothesis (Putnam, 1995), we tested if the absence of civic engagement with others is correlated with different attitudes toward specific characteristics. Therefore, we asked the participants if they had voted in the last election and whether they belonged to a social club (e.g., sports club). We further tested if participants’ political views correlate with distinct explicit attitudes toward some characteristics. The survey required participants to self-report their political views from extremely liberal to extremely conservative on a 7-point Likert scale. Demographic questions were not used as exclusion criteria.

### 2.3 Participants

We recruited participants through the crowdsourcing platform Amazon mTurk. We limited the possible participants to those who had a work approval rate of 80%, were based in the USA, were assigned the Master’s qualification, and had not completed a task for our lab previously. To exclude participants who had completed a task for our lab previously, we downloaded the list of participants in our tasks (HIT; Human Intelligence Tasks), extracted the unique worker ID, updated the list of participants, and assigned them an excluding qualification type in mTurk. Participants received $2 per response. Data were collected during 72 h in August 2023. The respondents remained anonymous throughout the experiment unless they contacted the research team for which they had to use e-mail.

### 2.4 Data analyses

#### 2.4.1 Scoring

Each of the 46 template narratives was manually curated with a scoring key that matched each of the possible responses. For example, for the following narrative: “*You had a terrible cab ride home from the airport, driven by an X. How much would you tip them? A. 0%, B. 10%, C. 20%*,” answer choice A was scored as 0 points, choice B as 0.5 points, and choice C as 1 point in favor of characteristic X.

For narratives that contained more than one agent, a scoring key for each character in the narrative was used. For example, for the following narrative: “*You’re a beggar on the streets of a major city. You see one person, who is an X about to walk by, and you see another person, who is a Y about to walk by. Who do you ask for money? A. First, B. Second*,” answer choice A is scored as 1 point for characteristic X and 0 points for characteristic Y, and answer choice B is scored as 0 points for characteristic X and 1 point for characteristic Y.

In this way, we were able to score decisions as favorable and unfavorable across a broad variety of naturalistic scenarios and social agents involved. The scoring of all narratives was transformed to the interval [0 1] in order to normalize the responses within an interval (see also Table 1). Normalizing the responses facilitated comparability between disparate questions and possible answers. While ordinal responses may not neatly map to a ratio answer, we have assumed that participants responded as if the options were ordinal. With the goal of increasing engagement in the task we provided a variety of response options, including ordinal (e.g., select 0, 10, 20% tip) and categorical (e.g., choose A over B).

#### 2.4.2 Statistics

We built a generalized linear mixed-effects model (LME) that included each category (gender, race, emotion, socioeconomic status) and their two-way interactions as fixed effects, and included the participant’s ‘identity’ and the narrative template number as random effects. For comparing the preference for one characteristic to another within one category, e.g., in the gender category: female vs. male, we conducted an independent samples *t*-test, after performing an equal variances test. If the test suggested unequal variances, we used Welch’s *t*-test. To investigate if there were statistical interactions across categories in three-characteristic narratives (e.g., black well-dressed male vs. white poorly dressed male), a 2-way ANOVA was performed. Lastly, paired samples *t*-test comparing in-group and out-group preferences for Race, Gender, and socioeconomic status were conducted to gauge the effect of in-group/out-group bias on a participant’s response. These were followed by estimating Cohen’s *D* to then calculate *post hoc* the statistic’s power using G*Power (Version 3.1.9.7; Faul et al., 2009).

We examined the possible relationship between distinct self-reported demographics and the evaluation of each characteristic using an LME. We included as fixed-effects variables the demographic variables that we had collected. Namely: age, education level, having voted in the previous election, membership in a social club, political leaning, gender, household income, and race. Race and gender were encoded as effects dummy variables. Thus, there are *n-1* dummy variables in these categories. At the same time, the participant’s ‘identity’ and the narrative template number were treated as random effects.

To assess *post hoc* the power of the LME model to estimate a true effect on narrative scores we proceeded as follows. We generated 1,000 replicates of synthetic data that reflected the study design, with 250 participants responding to 50 items. The percentage of items testing each characteristic per synthetic participant was similar to the original study (100% tested gender; ~60% tested race, emotion, dress, or an interaction between gender and race, emotion, or dress; ~25% tested an interaction between race, emotion, and dress). For simplicity, responses were binary. Synthetic responses had normal noise, while one fixed effect or interaction between parameters had a true effect of 0.05 (in narrative score units). We then fitted the LME model to the simulated data. Finally, to estimate the power of the LME, we calculated the proportion of simulations where the model correctly detects significant effects, using *α* = 0.05, for the interaction terms and fixed effects. The power of our approach was 100% for either fixed effects or interactions.

Political leaning had a significant effect on the overall participants’ responses, with increasing conservatism related to lower scores. To explore this relationship further we performed a partial correlation between narrative score and political leaning for each characteristic, controlling for all other characteristics. Throughout we set *α* = 0.05, report the uncorrected *p*-values, and correct for multiple comparisons using False Discovery Rate. Data analyses were performed on R (Version 4.1.1), SPSS (Version 27), and Matlab (Version 9.10).

## 3 Results

### 3.1 Responses to generative narrative survey and validation measures

We analyzed the responses of 257 US-based participants, recruited through mTurk, to a survey about attitudes toward four different categories of characteristics: gender, race, emotion, and socio-economic status. For convergent validity, we used two instruments to assess the participants’ attitudes: (1) a feeling thermometer and (2) a generative narrative survey. In the feeling thermometer, participants reported how warm they felt at that moment toward each characteristic using a feeling thermometer question *“Please rate how warm or cold you feel towards ~ insert characteristic here ~ people (0 = coldest feelings, 5 = neutral, 10 = warmest feelings).”* These feeling thermometer questions have been used to measure attitudes explicitly (Alwin, 1997), and responses to these items are positively correlated with other responses to other instruments. In the generative narrative survey, we presented brief scenarios in which agents with distinctive characteristics take an action and the participants play a role and are then asked to decide their course of action. Finally, to confirm that the participants indeed attended to the narratives, we asked comprehension questions in which the questions related to the narratives could be answered unambiguously from the information contained in the narrative.

**Figure 1:**
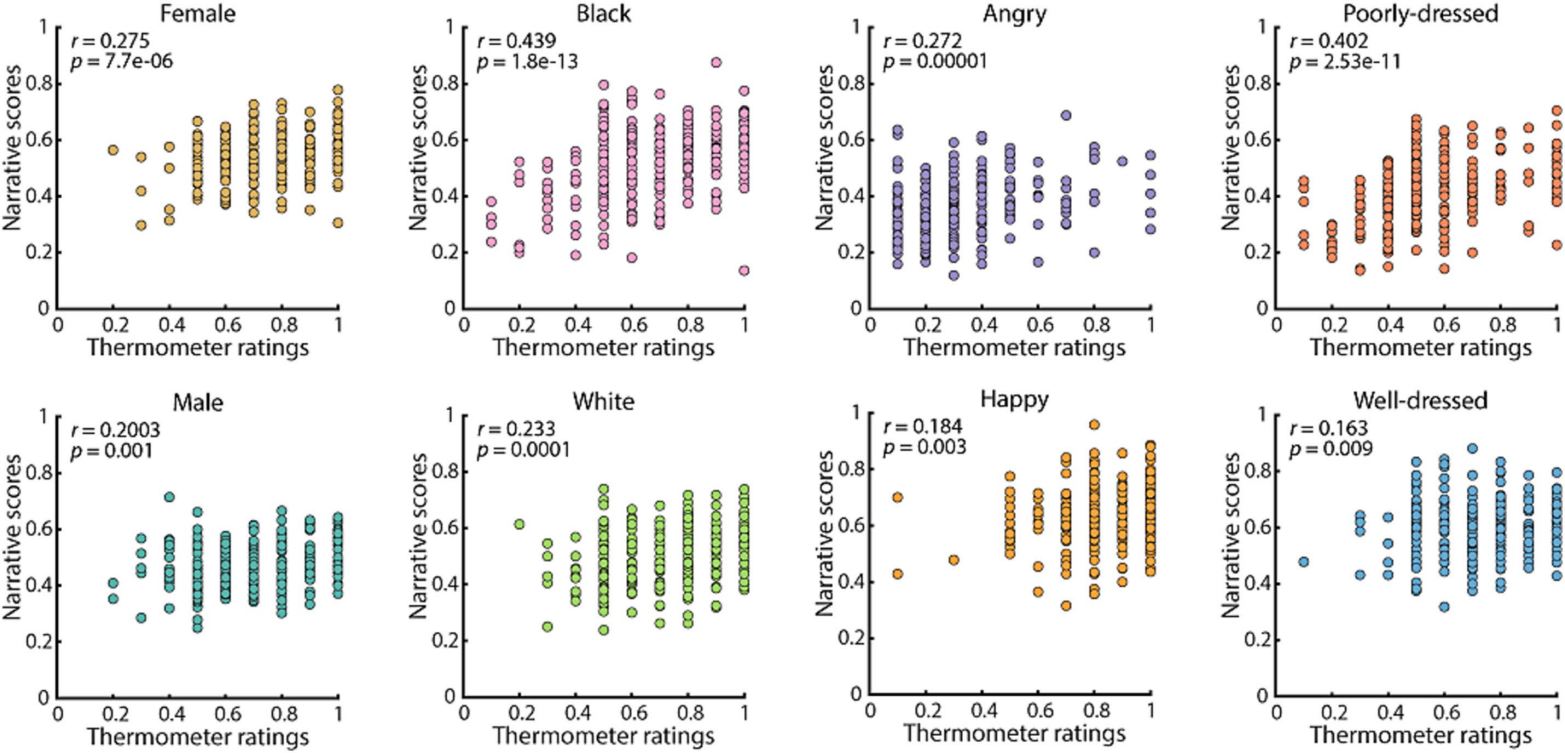
Convergent validity between thermometer ratings and narrative scores. Scatterplot of participant’s (*n =* 255) thermometer ratings and narrative scores for each characteristic. Each panel contains the Pearson correlation coefficient and the associated *p*-value between these two variables.

First, to confirm the convergent validity of the questionnaires, we examined the responses of the participants to the comprehension questions. Overall, of the 257 participants, we found that 255 (99.3%) displayed a comprehension performance above 60% and were therefore included in the remainder of the analyses (Table 3). Second, we confirmed that responses provided for the narrative questions as assessed through the narrative scores (0–1 with 0 being unfavorable, and 1 being favorable) correlated with their thermometer scores (with 0 being cold feelings and 1 being warm feelings) across the participants. Overall, participants felt more positive than negative about all characteristics (i.e., with feeling thermometer ratings >0.5), except for the adjective ‘angry’ [0.3239 ± 0.0125, mean ± standard error of the mean (SEM); *n =* 255, *t*(254) = 14.084, *p* = 1.108e-33, one-sample *t*-test with mean 0.5]. The participant’s responses to the same characteristics for the characters in the narratives showed a similar preference. This was true when considering all characteristics simultaneously (*r* = 0.523, *p* = 4.102e-14; Pearson correlation), or each one individually (Figure 1). Thus, the narratives elicited similar evaluations of each characteristic to asking participants how warm they felt toward each characteristic. Together this suggests that the participants’ answers to the narrative questions reflected their subjective judgments on a participant-by-participant level.

**Table 3:** Cross-tabulation of participants based on their reported gender, age, and race.

		*Gender*								
		*Other*			*Female*			*Male*		
		*Age Bracket*								
		*18–39*	*40–59*	*60–99*	*18–39*	*40–59*	*60–99*	*18–39*	*40–59*	*60–99*
*Race*	*Asian*	0	0	0	3	7	0	7	4	3
	*Hispanic*	0	0	0	0	1	1	6	3	0
	*Black*	0	0	0	5	7	1	3	4	0
	*White*	0	2	1	21	48	25	50	55	9

Multiple races could be selected.

**Figure 2:**
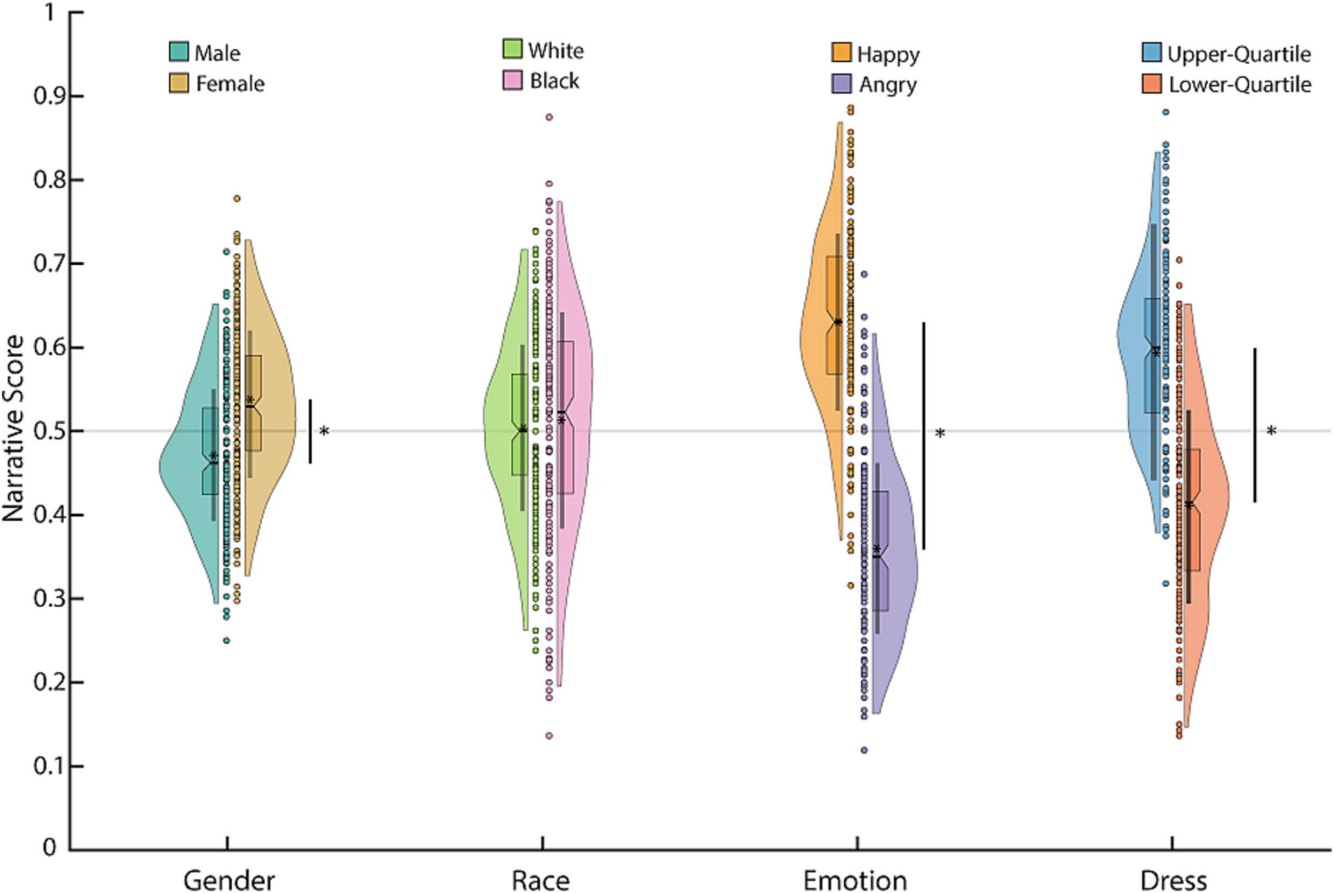
Narrative scores for each characteristic, grouped by characteristic category. Each circle is the mean narrative score of a participant; the boxplot shows the median, interquartile range, and the 95% confidence interval for the median. The asterisk in the boxplot denotes the mean and the line the mean ± 1 standard deviation. The distribution is a kernel density. Asterisks on the bar denote significant differences using an independent samples *t*-test, *p* < 0.05.

Across narratives, the choices of the participants were influenced by the social characteristics of the agents described in the narratives. After grouping characteristics by category, we discovered that participants showed a significant preference for one characteristic in gender, emotion, and dress (our proxy for socioeconomic status), but not for race. There was no significant difference in preference for ‘white’ versus’ black’ (*t*(508) = 0.890, *p* = 0.374; independent samples *t*-test), but participants significantly preferred ‘female’ over ‘male’ (*t*(508) = 8.203, *p* = 1.935e-15), ‘happy’ over ‘angry’ (*t*(507) = 29.405, *p* = 9.977e-112), and ‘well-dressed’ over ‘poorly-dressed’ (*t*(508) = 19.083, *p* = 1.304e-61) agents (Figure 2). Therefore, when taken together, we confirmed that the participants (1) comprehended the survey questions, (2) their responses were significantly influenced by the agents described in the narratives, and that (3) their responses corresponded with those elicited by the feeling thermometer measurement.

### 3.2 The mixed effects of social characteristics on decision making

Our approach to generating novel narratives combined with broad sampling allowed us to test the interaction of characteristic categories (gender, race, emotion, and dress). We considered all four categories simultaneously by building a generalized linear mixed-effects (LME) model that included each category and their interactions as fixed effects and included participants’ identities and the narrative template as random effects. Here, we observed that gender, emotion, and dress were captured by coefficients highly significantly different from zero (all *p* < 1e-32), while race was not (*p* = 0.22; Table 4). Moreover, among all two-way interactions, only the interaction between race and socioeconomic status tended toward a significant effect (*p* = 0.054, uncorrected). These results suggested that gender, socioeconomic status, and emotional state of an agent had independent effects on how participants decided.

**Table 4:** Main and interaction effects predicting narrative scores.

Predictor variable	Estimate	SE	*t*-stat	*p*-val	# observations
Gender	0.030	0.003	9.035	0.000	18,290
Race	0.005	0.004	1.212	0.226	11,013
Emotion	−0.136	0.004	−32.146	0.000	10,935
Dress	−0.090	0.004	−21.359	0.000	10,976
Gender: Race	−0.002	0.004	−0.401	0.688	11,013
Gender: Emotion	0.001	0.004	0.330	0.741	10,935
Gender: Dress	0.001	0.006	0.137	0.891	10,976
Race: Emotion	0.005	0.004	1.088	0.277	4,877
Race: Dress	−0.012	0.006	−1.923	0.054	4,911
Emotion: Dress	0.006	0.006	0.969	0.333	4,846
			*Adjusted R^2^*	0.163	
			*AIC*	22,355	
			*Log Likelihood*	−11,163	

LME with individuals as random effects and narrative templates as random effects using: *Score ~ 1 + cGender + cRace + Emotion + Dress + cGender:cRace + cGender:Emotion + cGender:Dress + cRace:Emotion + cRace:Dress + Emotion:Dress + (1|SubjectNum) + (1|NarrNum)*. Where *cGender*, *cRace*, *Emotion*, and *Dress* are the gender, race, emotion, and dress of the character in the narrative, respectively. *SubjectNum* is a number identifying the participant and *NarrNum* is a number identifying the template narrative used. Standard Error (SE), Akaike Information Criterion (AIC).

**Figure 3:**
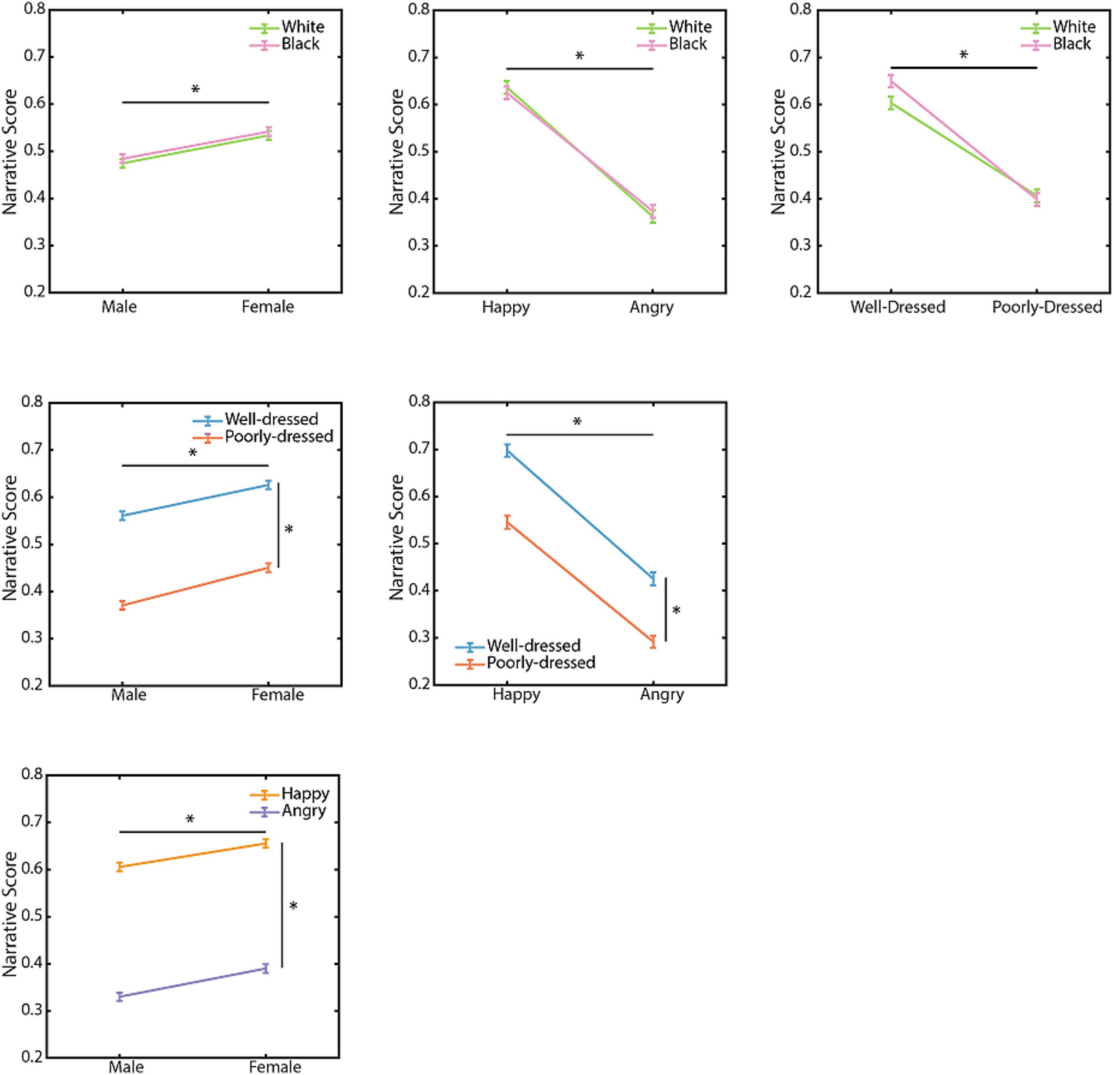
Narrative scores for combinations of characteristics. Line plots illustrate the relationship between narrative scores and distinct pairs of characteristics. Each point is the mean and error bars illustrate the standard error of the mean (SEM). The lines with an asterisk illustrate a significant difference in that factor, all *p* < 0.05, Two-way ANOVA.

Here, we tested the participant’s preferences for combinations of characteristics with a two-way ANOVA. For race and dress, there was an independent significant effect of dress, but not of race, while there was a significant interaction when not correcting for multiple comparisons using FDR [*F*(1, 4,907) = 278.450, *p* < 0.001, *Ω^2^* = 2.51%; *F*(1, 4,907) = 2.069, *p* = 0.150, *Ω^2^* = 0.01%; *F*(1, 4,907) = 4.030, *p* = 0.045, *Ω^2^* = 0.03%, respectively]. For race and gender, there was an independent significant effect of gender, but not of race and no significant interaction [*F*(1, 11,009) = 39.995, *p* < 0.001, *Ω^2^* = 0.17%; *F*(1, 11,009) = 0.893, *p* = 0.345, *Ω^2^* < 0.00%; *F*(1, 11,009) = 0.011, *p* = 0.917, *Ω^2^* < 0.00%, respectively]. For race and emotion, there was an independent effect of emotion but not of race or their interaction [*F*(1,4,873) = 384.393, *p* < 0.001, *Ω^2^* = 3.56%; *F*(1, 4,873) < 0.001, *p* = 0.987, *Ω^2^* < 0.00%; *F*(1, 4,873) = 0.691, *p* = 0.406, *Ω^2^* = −0.003%, respectively]. Both, emotion and gender showed a significant independent effect, but their interaction was not significant [*F*(1, 10,931) = 921.597, *p* < 0.001, *Ω^2^* = 3.8%; *F*(1, 10,931) = 37.849, *p* = 8e-10, *Ω^2^* = 0.15%; *F*(1, 10,931) = 0.318, *p* = 0.573, *Ω^2^* < 0.00%]. Similarly, both gender and dress showed a significant effect, but their interaction was not significant [*F*(1, 10,972) = 64.3, *p* = 1.18e-15, *Ω^2^* = 0.27%; *F*(1, 10,972) = 407.072, *p* = 6.4e-89, *Ω^2^* = 1.71%; *F*(1, 10,972) = 0.675, *p* = 0.411, *Ω^2^* = 2.51%]. Finally, both dress and emotion showed a significant effect but their interaction was not significant [*F*(1, 4,842) = 115.746, *p* = 1.08e-26, *Ω^2^* = 1.07%; *F*(1, 4,842) = 392.110, *p* = 5.5e-84, *Ω^2^* = 3.63%; *F*(1, 4,842) = 0.493, *p* = 0.483, *Ω^2^* < 0.00%; Figure 3]. Taken together, the emotional states of the agents therefore had the largest and most consistent effect on the participant’s choices. Further, whereas most characteristics had independent effects on decision making, the interaction of the agents’ perceived socioeconomic status and race affected the participant’s choices.

### 3.3 Social contrast effect

**Figure 4:**
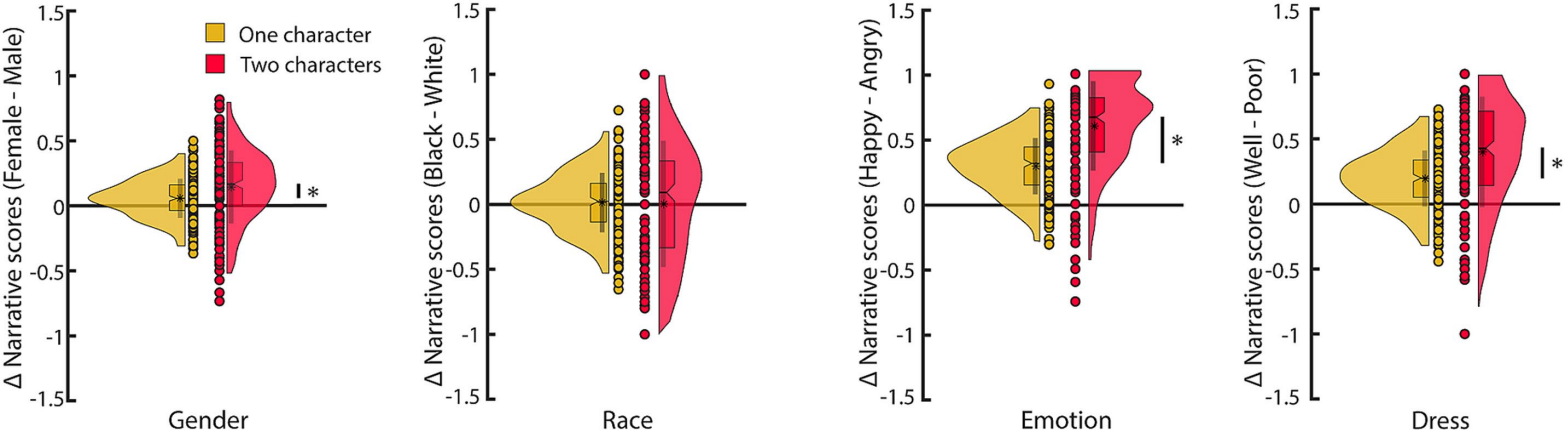
Social contrast effect in the difference in narrative scores when there was one character vs. two characters for each characteristic category. The *Y*-axis is the difference in narrative scores between the preferred and the non-preferred characteristics in each attribute category. Data in ocher shows the difference between the responses for the preferred vs. the least preferred characteristic when the narrative has only one character per participant. Data in red shows the difference between the average response when the narrative contains two characters. Each circle is a participant’s average narrative score; the boxplot shows the median, interquartile range, and the 95% confidence interval for the median. The asterisk in the boxplot denotes the mean and the line the mean ± 1 standard deviation. The distribution is a kernel density. The lines with an asterisk illustrate a significant difference (independent samples *t*-test, *p* < 0.05). *n =* 255 participants for each plot.

An advantage of the generative narrative survey approach is that it allows us to test participant’s preferences when one or two agents participate in the narrative. The effect of social characteristics on the participant’s choices was highly context-dependent. Dissonance theory (Festinger, 1954), for example, suggests that our judgments of others should be affected not only by their social characteristics but also by how those characteristics relate to other agents they may interact with. Therefore, to test this hypothesis, we calculated the difference in scores between characteristics of each category when there was only one character and in which there were two. First, we tested if the narrative scores for each character were higher than chance when the narratives had only one agent. The narrative scores of characters that were female, happy, and well-dressed were higher than chance [*t*(5164) = 3.39, *p* = 0.0006; *t*(3054) = 14.69, *p* = 6.36e-49; *t*(3147) = 11.43, *p* = 1.06e-29, respectively; one sample *t*-test against 0.5]. While the scores of male, angry, and poorly-dressed characters were significantly lower than chance [*t*(5201) = 5.94, *p* = 2.87e-9; *t*(3148) = 21.12, *p* = 1e-92; *t*(3068) = 12.75, *p* = 2.5e-36, respectively; one sample *t*-test against 0.5]. Neither the scores of Black or White characters were significantly different from chance [*t*(3050) = 0.62, *p* = 0.53; *t*(3123) = 1.15, *p* = 0.24, respectively; one sample *t*-test against 0.5]. We observed that when participants considered two agents in the gender, emotion, and socioeconomic status, there was a significant increase in their preferences compared to when only considering one [*t*(508) = 6.18, *p* = 1.251e-09; *t*(508) = 18.65, *p* = 1.592e-59; *t*(508) = 11.013, *p* = 1.922e-25, respectively]. Thus, for example, the participants were significantly more likely to favor a well-dressed male when that agent interacted with a poorly-dressed male than when considered in separate narratives. There were no significant changes in preference related to the race characteristics (*t*(508) = 0.569, *p* = 0.57; Figure 4). Therefore, the effect of all characteristics, aside from race, was significantly influenced by the social context of the agent’s interactions.

### 3.4 Demographic responses and ingroup and outgroup comparisons

We investigated how the participant’s own reported demographic characteristics influenced their responses. While we found variations in the participant’s responses based on their demographics and in particular their political leaning (Table 5), we examined the relationship between self-reported demographics and the evaluation of each characteristic using a LME model. We included as fixed-effects variables demographic variables that we had collected. Namely: age, education level, having voted in the previous election, membership in a social club, political leaning, gender, household income, and race. Race and gender were encoded as effects dummy variables. Thus, there were *n-1* dummy variables of these categories. At the same time, participant ‘identity’ and narrative template were treated as random effects. Here, we observed that only political leaning was a significant factor in the model (*p* = 0.973, *p* = 0.891 for gender-related coefficients; *p* = 0.138, *p* = 0.537, *p* = 0.226, *p* = 0.517, for race-related coefficients; *p* = 0.881, *p* = 0.183, *p* = 0.372, *p* = 0.184, *p* = 0.00008; age, education level, having voted in the previous election, membership in a social club, and political leaning, respectively; *t*-test on the estimated coefficient vs. null).

**Table 5:** Narrative scores for each characteristic by each aggregated demographic.

			Gender				Race				Emotion				Dress (Socioeconomic Status)			
			Female		Male		White		Black		Happy		Angry		Well-dressed		Poorly-dressed	
	Demographic	n	Mean	SEM	Mean	SEM	Mean	SEM	Mean	SEM	Mean	SEM	Mean	SEM	Mean	SEM	Mean	SEM
*Race*	*Asian*	24	0.522	0.016	0.448	0.016	0.478	0.015	0.514	0.021	0.621	0.017	0.325	0.019	0.584	0.018	0.381	0.024
	*Hispanic*	11	0.547	0.023	0.416	0.022	0.500	0.035	0.467	0.037	0.666	0.036	0.307	0.031	0.588	0.029	0.367	0.026
	*Black*	20	0.548	0.020	0.447	0.019	0.422	0.023	0.590	0.029	0.593	0.024	0.368	0.026	0.572	0.022	0.421	0.027
	*White*	211	0.532	0.006	0.478	0.005	0.514	0.007	0.510	0.009	0.635	0.007	0.363	0.007	0.598	0.007	0.414	0.008
*Gender*	*Other*	3	0.577	0.033	0.436	0.069	0.376	0.064	0.636	0.080	0.605	0.062	0.400	0.044	0.578	0.028	0.473	0.064
	*Female*	114	0.536	0.008	0.469	0.007	0.493	0.009	0.531	0.012	0.624	0.010	0.360	0.009	0.590	0.009	0.411	0.011
	*Male*	138	0.528	0.008	0.475	0.007	0.516	0.008	0.496	0.011	0.636	0.009	0.359	0.009	0.598	0.009	0.408	0.010
*House Income*	*Q1*	74	0.522	0.011	0.474	0.009	0.509	0.012	0.507	0.015	0.610	0.012	0.371	0.012	0.572	0.011	0.428	0.012
	*Q2*	106	0.541	0.009	0.469	0.008	0.506	0.010	0.513	0.013	0.642	0.010	0.361	0.010	0.601	0.010	0.411	0.012
	*Q3*	46	0.538	0.012	0.469	0.012	0.479	0.014	0.550	0.017	0.624	0.014	0.347	0.015	0.598	0.014	0.405	0.019
	*Q4*	29	0.519	0.015	0.480	0.016	0.524	0.015	0.469	0.025	0.647	0.023	0.349	0.019	0.622	0.020	0.372	0.019
*In Club*	*No*	197	0.534	0.006	0.474	0.006	0.509	0.007	0.515	0.009	0.638	0.008	0.361	0.007	0.593	0.007	0.414	0.008
	*Yes*	58	0.526	0.012	0.463	0.011	0.488	0.013	0.507	0.018	0.604	0.013	0.357	0.014	0.601	0.013	0.400	0.015
*Politics*	*Liberal*	39	0.566	0.013	0.480	0.014	0.488	0.017	0.576	0.017	0.665	0.016	0.364	0.016	0.578	0.019	0.470	0.015
	*2*	56	0.551	0.012	0.468	0.012	0.498	0.015	0.536	0.017	0.662	0.013	0.342	0.012	0.599	0.015	0.421	0.015
	*3*	45	0.527	0.011	0.477	0.010	0.497	0.012	0.528	0.017	0.630	0.017	0.355	0.014	0.592	0.017	0.409	0.019
	*4*	36	0.540	0.015	0.460	0.012	0.482	0.016	0.541	0.020	0.614	0.016	0.371	0.019	0.592	0.015	0.403	0.020
	*5*	34	0.507	0.016	0.464	0.012	0.530	0.017	0.445	0.021	0.580	0.020	0.387	0.018	0.586	0.015	0.388	0.020
	*6*	30	0.504	0.014	0.477	0.014	0.512	0.017	0.468	0.021	0.615	0.015	0.355	0.017	0.611	0.014	0.368	0.019
	*Conservative*	15	0.486	0.023	0.485	0.025	0.565	0.020	0.394	0.037	0.603	0.029	0.353	0.038	0.617	0.026	0.374	0.026
*Voted*	*No*	23	0.528	0.016	0.481	0.015	0.531	0.014	0.476	0.024	0.637	0.022	0.356	0.024	0.630	0.018	0.390	0.029
	*Yes*	232	0.533	0.006	0.471	0.005	0.501	0.007	0.517	0.009	0.630	0.007	0.360	0.007	0.591	0.007	0.412	0.007
*Education*	*High school*	34	0.522	0.015	0.452	0.011	0.509	0.018	0.490	0.024	0.611	0.016	0.355	0.017	0.563	0.018	0.402	0.017
	*Technical C.*	1	0.689	0.000	0.458	0.000	0.624	0.000	0.553	0.000	0.659	0.000	0.459	0.000	0.609	0.000	0.524	0.000
	*Some C.*	88	0.536	0.010	0.482	0.009	0.505	0.010	0.525	0.012	0.645	0.012	0.361	0.012	0.608	0.011	0.417	0.013
	*Bachelor*	101	0.528	0.008	0.468	0.007	0.501	0.010	0.510	0.013	0.626	0.010	0.354	0.009	0.590	0.010	0.409	0.012
	*Graduate S.*	31	0.541	0.016	0.476	0.015	0.504	0.018	0.511	0.025	0.623	0.019	0.379	0.020	0.605	0.015	0.403	0.021
*Age bracket*	*18–39*	78	0.529	0.011	0.465	0.010	0.499	0.011	0.502	0.016	0.630	0.013	0.353	0.011	0.594	0.013	0.407	0.013
	*40–59*	138	0.537	0.007	0.479	0.006	0.506	0.008	0.528	0.010	0.634	0.009	0.367	0.009	0.598	0.008	0.416	0.010
	*60–99*	39	0.522	0.015	0.458	0.012	0.508	0.018	0.484	0.022	0.619	0.015	0.350	0.015	0.583	0.016	0.398	0.020

Respondents could select multiple races. House income brackets are based on the US census, highest income bracket is Q4. Technical C. is Technical College, Some C. is Some College or Associate’s degree. Graduate S. is Graduate School.

**Figure 5:**
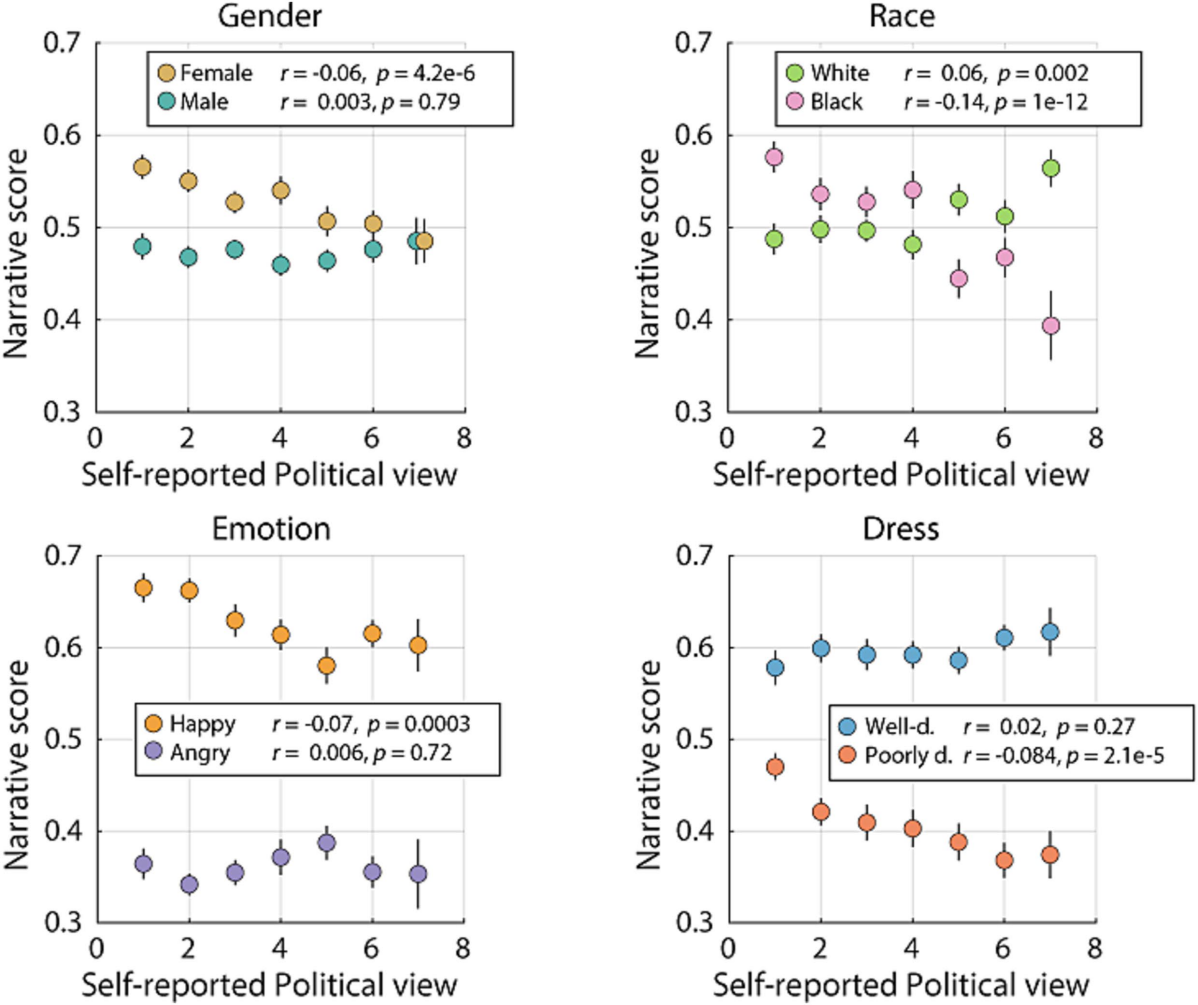
Relationship between political view and each characteristic. Scatter plot with means and SEM of the narrative score for each characteristic parsed by respondents’ self-reported political leaning, with higher numbers indicating being more conservative. *n =* [39, 56, 45, 36, 34, 30, 15], per level of self-reported political view. Partial correlations and associated *p*-values are included for each characteristic.

The survey required participants to self-report their political views from extremely liberal to extremely conservative on a 7-point Likert scale, with higher scores indicating more conservative views. The results from the LME model indicated that political leaning had a significant effect on the overall participants’ responses, with increasing conservatism related to lower narrative scores. To explore this relationship further we fitted a LME model that included political leaning, all main characteristics, and the two-way interactions between politics and each characteristic as fixed effects and the individual participants and narrative templates as random effects (Table 6). Here, we observed that increasingly conservative views were associated with lower scores for female, black, poorly-dressed, or happy characters. Correspondingly, increasing conservative views were associated with higher scores for male, white, richly-dressed agents, or happy characters. As a follow up to this result, we focused on measuring the relationship between political leaning and each characteristic, controlling for all other characteristics, using partial correlation. Here, we found a significant negative correlation between conservatism and the narrative scores of female, black, happy, and poorly dressed agents (*r* = −0.067, *p* = 4.25e-6; *r* = −0.1408, *p* = 1e-12; *r* = −0.0709, *p* = 3.43e-4; *r* = −0.084, *p* = 2.17e-5, respectively, Figure 5, partial correlation, *n =* 255 participants). Conversely, we found a positive correlation between conservatism and the narrative score of white agents (*r* = 0.061, *p* = 0.002, partial correlation, *n =* 255 participants). All other partial correlations were not significant (male: *r* = 0.003, *p* = 0.79; angry: *r* = 0.006, *p* = 0.72; richly-dressed: *r* = 0.02, *p* = 0.27). Therefore, political leaning influenced how the social characteristics of others affected the participants’ decisions.

**Table 6:** LME results after fitting the model.

Predictor variable	Estimate	SE	*t*-stat	*p*-value
Intercept	0.528	0.027	19.536	0
Politics	−0.007	0.002	−3.710	0.0002
Gender	0.050	0.007	7.086	1.43E-12
Race	0.067	0.009	7.366	1.84E-13
Emotion	−0.160	0.009	−17.564	0
Dress	−0.055	0.009	−6.070	1.30E-09
Politics: Gender	−0.006	0.002	−3.254	0.001
Politics: Race	−0.018	0.002	−7.664	1.89E-14
Politics: Emotion	0.007	0.002	2.943	0.003
Politics: Dress	−0.010	0.002	−4.346	1.39E-05
			Adjusted *R*^2^	0.1682
			AIC	22,249
			Log Likelihood	−11,111

*Score ~ 1 + Politics + cGender + cRace + Emotion + Dress + Politics:cGender + Politics:cRace + Politics:Emotion + Politics:Dress + (1|SubjectNum) + (1|NarrNum)* to the narrative scores of all participants.

**Figure 6:**
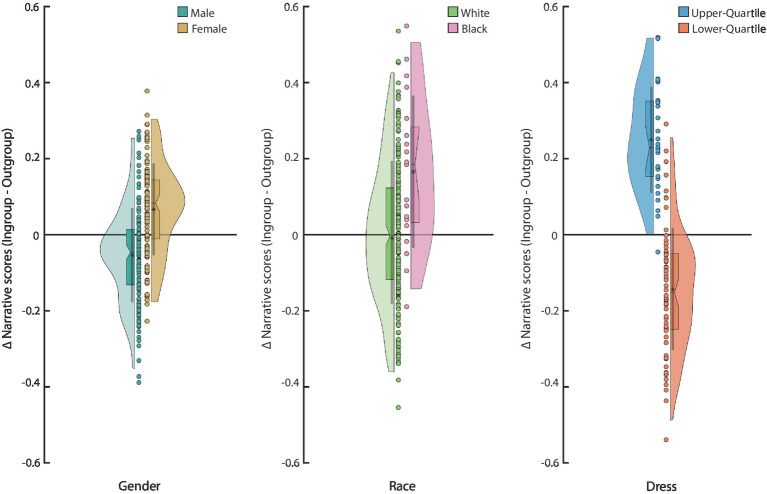
Ingroup favoritism is illustrated by the difference in narrative scores when the respondent could have considered the narrative characters as ingroup or outgroup based on their self-reported demographics. The *Y*-axis is the difference in narrative scores between the ingroup and outgroup for each demographic dimension: Male: *n =* 138, Female: *n =* 114; White: *n =* 210, Black: *n =* 19; Upper-income Quartile: *n =* 29; Lower-income Quartile: *n =* 74. The boxplot shows the median, interquartile range, and the 95% confidence interval for the median. The asterisk in the boxplot denotes the mean and the line the mean ± 1 standard deviation. The distribution is a kernel density.

Finally, we focused on the characteristics that could lead participants to perceive the characters in the narratives as belonging to either their ingroup or outgroup. Here, we find that both female and male participants preferred female agents [*t*(137) = 5.0828, *p* = 1.20e-06, *power* = 99%; *t*(113) = 5.9032, *p* = 3.81e-08, *power* = 100%, respectively; Paired samples *t*-test, Figure 6A]. While our main analyses indicate that there were no differences in preferences regarding the race of the agents (Figures 2–4), we found significant differences in the ingroup preferences. Specifically, black respondents showed an ingroup preference (*t*(18) = 3.59, *p* = 0.00207, *power* = 90.9%, ingroup vs. outgroup; Paired samples *t*-test), while white respondents did not show a preference for either group (*t*(209) = 0.3945, *p* = 0.69359, *power* = 6.0%; Paired samples *t*-test, Figure 6B). Lastly, respondents in both the upper quartile (*t*(28) = 9.63, *p* = 2.19e-10, *power* = 100%; Paired samples *t*-test) and the lower quartile (*t*(73) = 7.7372, *p* = 4.38e-11, *power* = 100%; Paired samples *t*-test) of household income preferred agents that were well dressed over those that were poorly dressed (Figure 6C).

## 4 Discussion

We designed a generative narrative survey in which we permuted distinct social characteristics from four distinct classes in many different interactive contexts to test simultaneously several hypotheses. Explicit and implicit attitudes regarding gender, race, and socioeconomic status have been extensively studied (Charlesworth and Banaji, 2019; Cunningham and Zelazo, 2007; Gilberstadt et al., 2020; Navajas et al., 2019; Pratto et al., 1994; Stanley et al., 2011). At the same time, it has long been acknowledged that other’s emotions have a strong impact on how we evaluate distinct situations (Klüver and Bucy, 1939; Papez, 1937; Sadedin et al., 2023; Zych and Gogolla, 2021). Yet, we need to learn more about how our decisions are affected by the interaction of distinct social characteristics of others within real-world scenarios (e.g., offering a tip to a poorly-dressed black male), their comparison (e.g., a poorly-dressed black male vs. a well-dressed white female), or the observer’s demographics.

Here, we found that gender, perceived socioeconomic status, and emotion but not race had a significant influence on how participants rated fictional agents. More notably, when evaluating two agents with different characteristics, participants’ preferences were stronger compared to only one character for the same categories in gender, perceived socioeconomic status, and emotion, but not race. While our participants preferred females over males and well-dressed over poorly dressed agents, regardless of their demographic characteristics, only black respondents showed ingroup favoritism. Finally, we identified a robust negative correlation between self-reported conservative political views and the narrative scores of distinct social characteristics: including being female, black, happy, and poorly dressed. We also found a positive correlation between conservatism and the narrative score of white agents; together revealing a remarkably detailed interrelationship between the effects of social characteristics, context, and sociodemographics on decision making.

In our panel, participants reported more positive feelings toward females than males and decided in favor of females over males. Past research indicates that attitudes toward females are more positive than those toward males (Eagly and Mladinic, 1994). While our instruments do not directly test prejudices or stereotypical behaviors, this positive evaluation might derive from stereotypical attitudes (Amodio and Cikara, 2021; Eagly and Mladinic, 1994). This deferential behavior is commonly expressed across cultural settings but varies by individual sociodemographic characteristics (Kågesten et al., 2016). Relatedly, we discovered that participants reporting more conservative viewpoints showed less positive views of females than liberal participants did. Furthermore, while boys and girls show ingroup preferences early in development, as males mature they show a preference for females (Dunham et al., 2016). Similarly, we did not find evidence of own-gender preferences in our panel of adult US-based participants. Thus, our results confirm the widely observed preference for females over males.

We tested attitudes toward two basic emotions with different valences: happiness and anger emotions (Tracy and Randles, 2011). Both happiness and anger are emotions that are relevant to the perceiver, and they can trigger approach and avoidance reactions (Paulus and Wentura, 2016). In line with previous findings, we observed that participants in our panel evaluated favorably happy over angry agents. Furthermore, these two emotions can be considered to be certain, vs. emotions associated with uncertainty like hopefulness and anxious (Tiedens and Linton, 2001). The certainty of happiness and anger might facilitate their judgment in others’ behaviors as observed in the large difference in narrative scores we observed. Similarly, a happy face may signal a wish for affiliation, and an angry face a wish to attack (Hess and Thibault, 2009), the context in which an emotion is perceived matters (Barrett et al., 2011). In our vignettes, inspired by real-life situations, participants showed a robust preference for happy agents over angry ones. Overall, these results underscore the robustness of our novel approach.

Overall, we found little evidence in favor of a strong preference based on race. This finding is in line with contemporary studies of attitudes toward race (Charlesworth and Banaji, 2019). The absence of differences in race may relate to a sensitive domain that elicits social desirability bias (An, 2015) that can be better predicted with other methods, like the implicit association test (Greenwald and Banaji, 1995; Kurdi et al., 2019a; Kurdi et al., 2019b). This absence might reflect the responders’ attitudes –as also shown in the feeling thermometer. On the other hand, black responders showed ingroup favoritism for black agents, while no other ingroup favoritism was observed. However, we are also cautious about strong inferences based on this result due to the relatively low number of self-reported black respondents.

Generally, people of higher socioeconomic status receive preferential treatment (Lott and Saxon, 2002). Sociodemographics, like political leaning, also influence how rich people are perceived with liberals less supportive of richer people (Parker, 2012). However, implicit rather than explicit biased attitudes toward the upper class are usually expressed (e.g., Horwitz and Dovidio, 2017). With the generative narrative survey, we found that across our sample-encompassing all family income quartiles-participants decided positively in favor of richly dressed characters. Furthermore, we observed a trend in higher narrative scores for black and richly dressed compared to white and richly dressed characters. While not statistically significant, we speculate that it might relate to a combination of prejudices and values commonly observed in the United States, from where we collected the data. On one hand, generally, people in the United States would like to be rich, the so-called American dream; and this value is also imbued with the concept of meritocracy, in which anyone can be rich if they have the merits (Kasser and Ryan, 1993). On the other, generally, people in this country have an implicit negative bias toward black people (Charlesworth and Banaji, 2019; Kubota et al., 2012; Stanley et al., 2011). Thus, we speculate that higher narrative scores for richly dressed black characters might be related to characters that achieved a widely held positive value. This finding and the associated hypotheses deserve further investigation.

Enhanced responses when contrasting two agents may relate to cognitive dissonance theory, in which we show preferences for attitudes that are consonant with our beliefs and attitudes (Egan et al., 2007). In particular, it suggests that the effect of social characteristics on decision making does not manifest in isolation but is rather strongly influenced by their social context. These findings also demonstrate the disparate roles that specific characteristics play and highlight the powerful influence that the behavior and emotional states of others have on our decisions.

Finally, in line with population-based surveys (Gilberstadt et al., 2020) we found that participants who report more conservative political leaning tend to evaluate black agents less favorably and to a lesser extent lower socioeconomic status, happy and more favorably white agents. It also demonstrates the variable effects that one’s demographics play when considering in vs. out-group characteristics.

Altogether, the convergent validity of the generative narrative survey with the feeling thermometer ratings, which have been shown to correlate with other measures of explicit attitudes (Axt, 2018; Payne et al., 2008), provides a novel approach for investigating explicit attitudes. Thus, it is likely that the evaluations in both the feeling thermometer ratings and the survey were conscious, effortful, and involved critical thinking (Morewedge and Kahneman, 2010). These evaluations correspond to explicit attitudes, in contrast to implicit attitudes. One intriguing possibility is to constrain the response time to probe the role of fast, automatic, and intuitive cognitive processing in these evaluations and correlate them with implicit measures of attitudes. While the generative narrative survey is designed to limit the participants’ response choices, two aspects should be taken into account in future studies. First, users should control for possible social desirability bias in how participants report their choices. Second, users should carefully design the number of options in the answers as these are not necessarily treated as ordinal options by the participants and users should consider increasing them to 10 or more options to strengthen their studies (e.g., Leung, 2011; Simms et al., 2019). The generative narrative survey approach can be flexibly used to test single or multiple characteristics. Analytical consideration should be taken into account when testing multiple characteristics. An important step in using the Generative Narrative approach in future studies is to simulate plausible results in order to set all experimental design parameters appropriately *a priori* (e.g., Lakens and Caldwell, 2021). Another possibility when using this approach is to re-use specific templates for contrasting participants’ preferences, although it should be considered that participants might read the narratives with less attention as they will be more similar and might not notice the subtle differences between them. Here, our approach efficiently tested multiple attitudes explicitly, and showed robust convergent validity, and revealed that respondents exhibited contrast effects. The generative narrative method also has the potential to be useful in obtaining attitude correlates in physiological testing contexts in which single-participant testing is time-restricted.

## Data Availability

The raw data supporting the conclusions of this article will be made available by the authors, without undue reservation.
